# Pediatric opioid and cannabis poisoning hospitalizations in the United States, 2016–2022: a national age-matched inpatient analysis

**DOI:** 10.3389/fped.2026.1904477

**Published:** 2026-09-03

**Authors:** Linor Fournier, Shereen Shehadeh, Mohamad Hamad Saied, David Maman, Giora Pillar

**Affiliations:** 1 Department of Pediatrics, Carmel Medical Center, Haifa, Israel; 2 The Ruth and Bruce Rappaport Faculty of Medicine, Technion - Israel Institute of Technology, Haifa, Israel; 3 Department of Pediatric Immunology and Rheumatology, Wilhelmina Children's Hospital, University Medical Center Utrecht, Utrecht, Utrecht, Netherlands

**Keywords:** cannabis poisoning, health care utilization, hospitalization, national inpatient sample, opioid poisoning, pediatric poisoning, respiratory failure

## Abstract

**Objective:**

Pediatric poisonings involving opioids and cannabis represent two evolving public health challenges. This study compared national inpatient outcomes, complications, and hospital charges among children and adolescents hospitalized with a principal diagnosis of opioid vs. cannabis poisoning in the United States.

**Methods:**

We conducted a retrospective cohort study using the Healthcare Cost and Utilization Project National Inpatient Sample from 2016 to 2022. Children and adolescents aged 0–18 years with a principal diagnosis of opioid or cannabis poisoning were identified using ICD-10-CM codes. National estimates were generated using the NIS discharge weight (DISCWT), with variance estimation accounting for NIS_STRATUM and HOSP_NIS. A 1:1 nearest-neighbor age-matched analysis was performed on the unweighted discharge records before application of the national weights. Survey-weighted logistic regression was used for binary outcomes, and survey-weighted linear regression was used for length of stay and total hospital charges. Multivariable models were adjusted for age, sex, race**,** median household income quartile, weekend admission, and admission year.

**Results:**

The study included 17,555 weighted pediatric hospitalizations, comprising 10,780 opioid-related and 6,775 cannabis-related poisonings. The proportion classified as cannabis poisoning increased from 24.0% in 2016 to 47.2% in 2022 (survey-weighted trend odds ratio per year, 1.18; 95% CI, 1.14–1.23; *P* < 0.001). In the age-matched analysis, opioid poisoning was associated with higher risks of mechanical ventilation for more than 24 h (RR, 3.15; 95% CI, 2.00–4.95), acute kidney injury (RR, 1.58; 95% CI, 1.10–2.25), and respiratory failure (RR, 2.33; 95% CI, 1.25–4.35). In the full multivariable analysis, opioid poisoning remained associated with mechanical ventilation for more than 24 h (aOR, 4.65; 95% CI, 2.96–7.31) and acute kidney injury (aOR, 2.55; 95% CI, 1.77–3.69). Opioid poisoning was also associated with an adjusted increase of 1.06 hospital days (95% CI, 0.78–1.34) and $17,286 in hospital charges (95% CI, $12,752-$21,820). Mortality was not reported comparatively because the unweighted number of events in one exposure group was 10 or fewer.

**Conclusion:**

Cannabis poisoning accounted for an increasing proportion of pediatric opioid and cannabis poisoning hospitalizations during the study period. Nevertheless, opioid poisoning was associated with greater respiratory morbidity, longer hospitalization, and higher hospital charges. These findings support opioid-related respiratory monitoring and harm-reduction efforts by clinicians and public health agencies, together with child-resistant packaging, safe-storage counseling, and caregiver education for cannabis products.

## Introduction

Pediatric poisoning is an important cause of preventable morbidity, hospitalization, and health care utilization (1–4). Children and adolescents are vulnerable to toxic exposures for different reasons across developmental stages. Young children are particularly susceptible to unintentional ingestion because of exploratory behavior and access to medications or other substances in the home, whereas adolescents may experience poisoning in the setting of intentional substance use, misuse, or self-harm (3, 4). Psychoactive substances are of particular concern because severe exposures may result in central nervous system depression, respiratory compromise, and intensive care utilization (1–4).

Among pediatric poisonings, opioids represent a particularly severe exposure category. The opioid crisis, initially recognized primarily as an adult public-health emergency, has increasingly affected children and adolescents. Between 2004 and 2015, pediatric intensive care unit admissions for opioid ingestions more than doubled, with nearly 40% of cases requiring mechanical ventilation and mortality rates approaching 1.6% (1). Broader critical care data have similarly demonstrated the substantial resource burden associated with opioid overdose (2). National trend data also demonstrated a 165% increase in pediatric opioid poisoning hospitalizations between 1997 and 2012 (3). In addition, a large analysis of more than 200,000 pediatric opioid ingestions from the National Poison Data System highlighted the vulnerability of very young children and the substantial health care burden associated with these exposures (4).

Cannabis-related pediatric exposures have also increased, particularly with legalization, commercialization, and the availability of edible cannabis products. Pediatric edible cannabis exposures increased by more than 1,300% between 2017 and 2021, with most cases presenting with central nervous system depression and nearly one quarter requiring hospitalization (5). Additional studies have shown that edible cannabis exposures in children are frequently associated with prolonged symptoms, emergency evaluation, and intensive monitoring or admission (6, 7). Recent surveillance reports have further emphasized rising cannabis-related pediatric hospitalizations, particularly among young children (8, 9). International data have also shown increases in cannabis poisoning hospitalizations after legalization and the introduction of edible products (10). These trends are especially concerning because cannabis-infused edible packaging may contain features that appeal to children and adolescents (11).

Despite these parallel trends, opioids and cannabis are rarely compared directly in hospitalized pediatric populations. Most prior studies have examined each substance separately, relied on poison-center data rather than inpatient hospitalization data, or lacked adjustment for important differences between exposed populations, particularly age (1–11). As a result, the relative inpatient severity, complication profile, and economic burden of pediatric opioid vs. cannabis poisoning remain incompletely defined at the national level.

Therefore, using the Healthcare Cost and Utilization Project National Inpatient Sample from 2016 to 2022, we compared pediatric hospitalizations with a principal diagnosis of opioid poisoning vs. cannabis poisoning. We evaluated national trends, age-specific patterns, length of stay, hospital charges, and major inpatient complications. Because age differed substantially between the exposure groups, we performed an age-matched analysis. We additionally used survey-weighted multivariable logistic regression for binary outcomes and survey-weighted linear regression for length of stay and hospital charges. We hypothesized that, despite the increasing proportion of cannabis-related hospitalizations, opioid poisoning would remain associated with greater inpatient morbidity and hospital resource utilization.

## Materials and methods

### Data source

We conducted a retrospective cohort study using the Healthcare Cost and Utilization Project (HCUP) National Inpatient Sample (NIS) for 2016–2022. The NIS is a nationally representative all-payer database of inpatient discharges in the United States and includes discharge weights, sampling strata, and hospital cluster identifiers. The NIS was selected because the study objective required annual estimates across seven consecutive years. The pediatric-specific Kids' Inpatient Database was not used because it was available for only 2016, 2019, and 2022 during the study period and therefore could not support annual trend analysis. National estimates were generated using the NIS discharge weight, and variance estimation accounted for the stratified and clustered sampling design.

### Study population and case definition

Children and adolescents aged 0–18 years with a principal ICD-10-CM diagnosis of opioid or cannabis poisoning were included. Opioid poisoning was identified using T40.0× (opium), T40.1× (heroin), T40.2× (other opioids), T40.3× (methadone), T40.4× (other synthetic narcotics, including fentanyl), and T40.6× (other and unspecified narcotics). Cannabis poisoning was identified using T40.7× (cannabis and cannabis-related compounds).

Exposure classification was based on the principal diagnosis. Hospitalizations with an eligible opioid code as the principal diagnosis were assigned to the opioid group, whereas hospitalizations with T40.7× as the principal diagnosis were assigned to the cannabis group. Secondary diagnosis codes were not used to reassign the principal exposure group.

### Demographic and hospital variables

The primary exposure was poisoning type, categorized as opioid poisoning or cannabis poisoning. Age, length of stay, and total hospital charges were analyzed as continuous variables. Sex and weekend admission were analyzed as binary variables. Race was analyzed as a categorical variable using the standard HCUP categories of White, Black, Hispanic, Asian or Pacific Islander, Native American, and Other. Median household income for the patient's ZIP code was categorized into national quartiles: 0–25th, 26th–50th, 51st–75th, and 76th–100th percentile. Admission year was analyzed as a categorical covariate in the multivariable outcome models and as a continuous variable in the temporal trend analysis.

### Clinical and economic outcomes

The primary resource-utilization outcomes were length of stay, measured in days, and total hospital charges, measured in US dollars. Hospital charges represent amounts billed by hospitals and should not be interpreted as actual costs or reimbursements.

The evaluated clinical outcomes included in-hospital mortality, respiratory failure, mechanical ventilation for more than 24 h, mechanical ventilation for more than 96 h, acute respiratory distress syndrome, sepsis, pneumonia, acute kidney injury, and urinary tract infection. Complications and mechanical ventilation were identified using ICD-10-CM diagnosis codes and ICD-10-PCS procedure codes, respectively.

Mortality and other outcomes for which an exposure-specific unweighted event count was 10 or fewer were not reported comparatively and were not included in regression models, in accordance with HCUP reporting requirements.

### Age matching and cohort balancing

Because patients with opioid poisoning were older on average than those with cannabis poisoning, we performed 1:1 nearest-neighbor age matching using a maximum caliper of one year and without replacement. Matching was performed on the unweighted discharge-level records before application of the NIS discharge weights. Hospitalizations without an eligible age match were excluded from the matched analysis but remained in the full-cohort analysis. The inability to match all cannabis hospitalizations primarily reflected a relative shortage of eligible opioid comparators at several mid-childhood ages. Post-match age balance was evaluated using the standardized mean difference, with an absolute value below 0.10 considered acceptable. The post-match standardized mean difference for age was 0.009, indicating excellent balance.

### Statistical analysis

All national estimates incorporated the NIS discharge weight (DISCWT). Variance estimation accounted for the complex NIS sampling design using NIS_STRATUM as the stratification variable and HOSP_NIS as the hospital cluster variable. For pooled multiyear analyses, calendar year was incorporated into the stratum and cluster identifiers. Taylor-series linearization was used to estimate standard errors.

Continuous variables were summarized using weighted means and standard deviations, and categorical variables were summarized using weighted frequencies and percentages. Between-group comparisons were performed using design-adjusted tests for continuous variables and Rao-Scott design-adjusted chi-square tests for categorical variables.

The temporal change in poisoning type was evaluated using survey-weighted logistic regression, with poisoning type as the dependent variable and calendar year entered as a continuous predictor. The resulting odds ratio represents the annual change in the odds that an included hospitalization was classified as cannabis rather than opioid poisoning.

The age-matched analysis was conducted using hospitalizations matched on the unweighted discharge records, after which the NIS discharge weights were applied. Relative risks with 95% confidence intervals were calculated for outcomes with more than 10 unweighted events in each exposure group. Outcomes not meeting this reporting threshold were suppressed.

Unadjusted regression models first estimated the crude association between poisoning type and each reportable outcome. Multivariable regression analyses were then performed in the full unmatched cohort. Binary outcomes were analyzed using survey-weighted logistic regression and are reported as odds ratios with 95% confidence intervals. Length of stay and total hospital charges were analyzed using survey-weighted linear regression and are reported as beta coefficients with 95% confidence intervals. Cannabis poisoning was the reference exposure. Multivariable models were adjusted for age, sex, race, median household income quartile, weekend admission, and admission year.

Hospital charges were retained on their original dollar scale because the prespecified estimand was the absolute difference in hospital charges between the exposure groups, reflecting the national hospital charge burden. The adjusted coefficient therefore represents the absolute difference in hospital charges in US dollars.

Missing data were not imputed. Descriptive analyses used all available observations, whereas regression models used complete-case analysis for the variables included in each model. Statistical significance was defined as a two-sided *P* value <0.05. Analyses were performed using SPSS Complex Samples procedures.

### Ethical considerations

This study used fully de-identified discharge-level data and involved no direct patient contact or identifiable private information. The study was reviewed by the Carmel Medical Center Helsinki Committee, Haifa, Israel, which issued a written exemption from formal ethical approval. No reference number was assigned to the exemption. The requirement for informed consent was waived because the study used de-identified administrative data. The study was conducted in accordance with applicable institutional requirements and the principles of the Declaration of Helsinki.

## Results

### Trends in pediatric poisoning from Cannabis and opioids (2016–2022)

**Figure 1:**
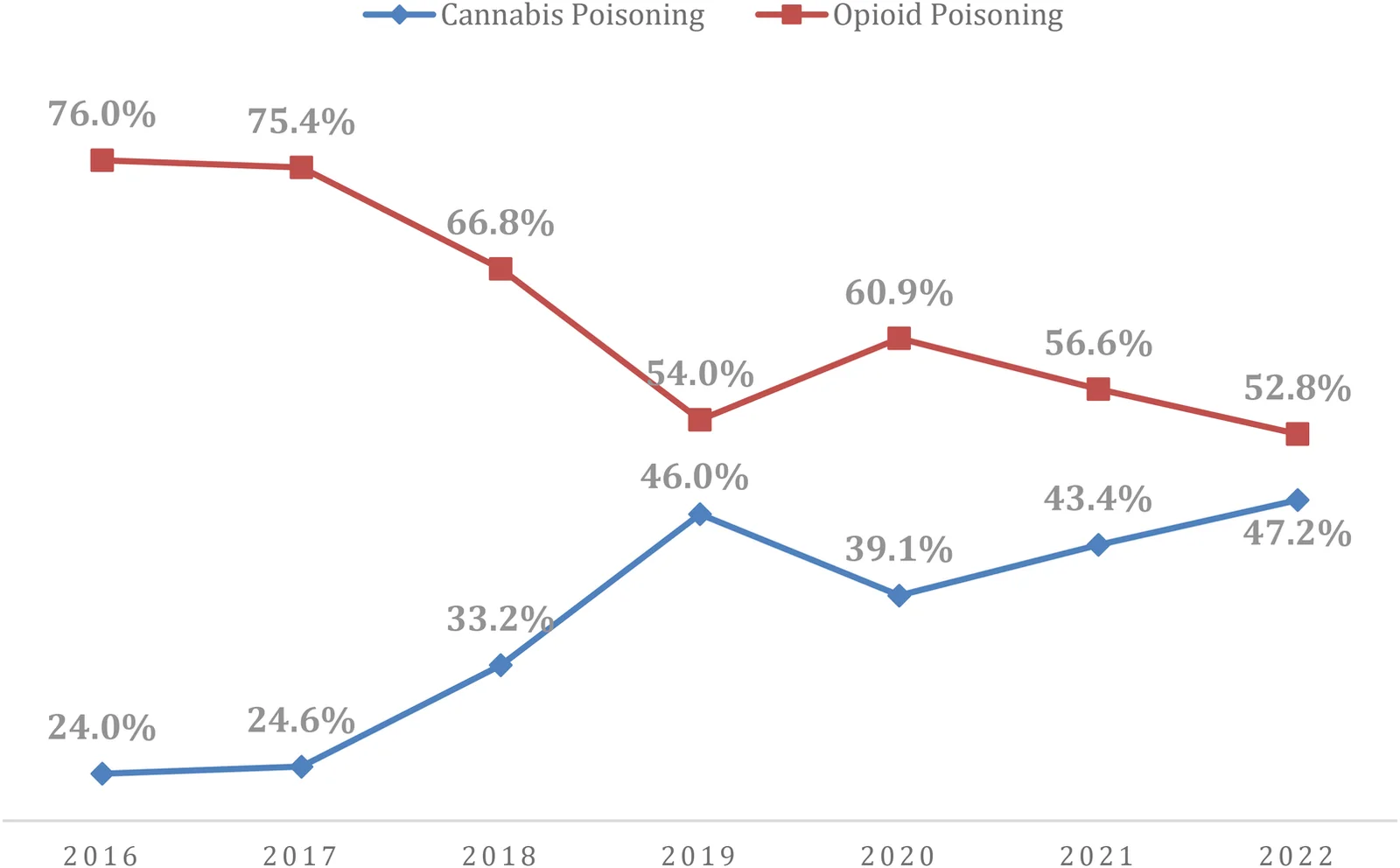
Annual proportion of pediatric opioid and cannabis poisoning hospitalizations, 2016–2022.

Figure 1 presents the annual distribution of pediatric opioid and cannabis poisoning hospitalizations. The proportion classified as cannabis poisoning increased from 24.0% in 2016 to 47.2% in 2022, whereas the proportion classified as opioid poisoning decreased from 76.0% to 52.8%. Survey-weighted logistic regression demonstrated a significant overall annual increase in the odds of cannabis rather than opioid classification (OR per year, 1.18; 95% CI, 1.14–1.23; *P* < 0.001). The change was not monotonic in every individual year; for example, the cannabis proportion decreased from 46.0% in 2019 to 39.1% in 2020 before increasing again. Accordingly, the findings indicate a significant overall temporal trend with year-to-year variation.

### Age distribution of pediatric poisonings by substance type

**Figure 2:**
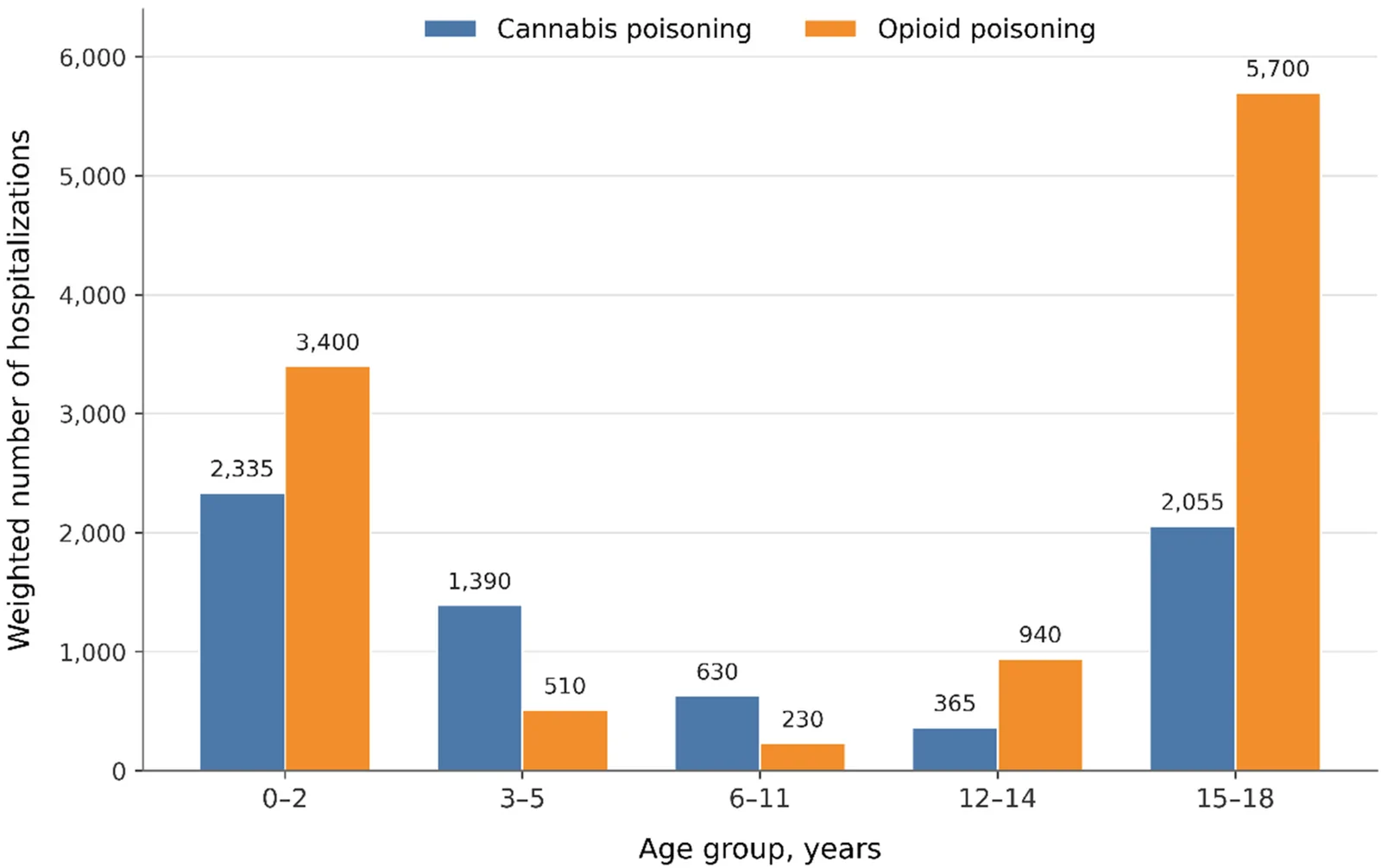
Weighted number of pediatric opioid and cannabis poisoning hospitalizations by age group.

Figure 2 presents the weighted number of opioid and cannabis poisoning hospitalizations by age group. Two major age-related patterns were observed. Among children aged 0–2 years, there were approximately 3,400 opioid and 2,335 cannabis poisoning hospitalizations. During mid-childhood, cannabis poisoning was more frequent, with approximately 1,390 vs. 510 hospitalizations among children aged 3–5 years and 630 vs. 230 hospitalizations among those aged 6–11 years. In adolescence, opioid poisoning again predominated, particularly among patients aged 15–18 years, with approximately 5,700 opioid vs. 2,055 cannabis poisoning hospitalizations.

### Sociodemographic patterns of pediatric hospitalizations Due to Cannabis and opioid poisoning

Table 1 presents the sociodemographic characteristics of the study population. The analysis included 17,555 weighted hospitalizations, comprising 10,780 opioid-related and 6,775 cannabis-related poisonings. Patients in the opioid group were older than those in the cannabis group (mean age, 10.7 vs. 7.7 years; *P* < 0.001). Sex distribution and weekend admission did not differ significantly between the groups. The overall distributions of race and median household income quartile differed significantly by poisoning type (both *P* < 0.001). Opioid poisoning hospitalizations included a higher proportion of White patients, whereas cannabis poisoning hospitalizations included higher proportions of Black and Hispanic patients. Race was missing for 223 unweighted hospitalizations (6.4%), and median household income quartile was missing for 40 hospitalizations (1.1%). Missing values were not imputed.

**Table 1:** Sociodemographic characteristics of pediatric hospitalizations with a principal diagnosis of opioid or cannabis poisoning.

Parameter	Opioid Poisoning	Cannabis Poisoning	Significance
Total Cases	10,780	6,775	–
Average Age (y)	10.7	7.7	*P* < 0.01
Female (%)	47.2	47.5	*P* = 0.89
Admission day is a weekend (%)	29.8	28.7	*P* = 0.49
Race—White (%)	56.2	38.3	*P* < 0.01
Race—Black (%)	16	27.7	
Race—Hispanic (%)	20	24.9	
Race—-Asian or Pacific Islander (%)	1.3	1.3	
Race—Native American (%)	1.2	1.7	
Race—Other (%)	5.3	6.2	
Median household income—0–25th percentile	31.6	38.5	*P* < 0.01
Median household income—26th–50th percentile	26.2	25.3	
Median household income—51st–75th percentile	23.2	21	
Median household income—76th–100th percentile	18.9	15.1	

Values are survey-weighted national estimates. *P* values for race and median household income represent overall design-adjusted tests across all categories. Percentages were calculated using available data; missing values were not imputed.

### Clinical and economic outcomes of pediatric Cannabis vs. Opioid Poisoning

Table 2 compares hospital resource utilization between the exposure groups. Opioid poisoning was associated with a longer mean hospital stay than cannabis poisoning (2.9 vs. 1.6 days; *P* < 0.001). Mean hospital charges were also higher in the opioid group ($38,680 vs. $20,600; *P* < 0.001). Comparative mortality results were not reported because the unweighted event count in one exposure group was 10 or fewer.

**Table 2:** Hospital resource utilization in pediatric opioid and cannabis poisoning hospitalizations.

Parameter	Opioid poisoning	Cannabis poisoning	Significance
Length of stay mean in days	2.9 (Std. deviation 5.6)	1.6 (Std. deviation 1.7)	*P* < 0.01
Total charges mean in $	38,680 (Std. deviation 94,215)	20,600 (Std. deviation 23,098)	*P* < 0.01

Values are survey-weighted national estimates. Hospital charges represent amounts billed and not actual costs or reimbursements.

### Matched cohort analysis: clinical and economic outcomes of pediatric Cannabis vs. Opioid Poisoning

A total of 1,153 unweighted opioid poisoning hospitalizations were matched to 1,153 cannabis poisoning hospitalizations. After application of the NIS discharge weights, each group represented approximately 5,765 national hospitalizations. Matching was performed on the unweighted discharge records before application of the national weights. The mean age was 8.1 years in both groups, and the standardized mean difference was 0.009, indicating excellent age balance.

In the matched cohort, opioid poisoning was associated with a longer mean hospital stay than cannabis poisoning (2.5 vs. 1.7 days; *P* < 0.001) and higher mean hospital charges ($29,032 vs. $21,364; *P* = 0.001). Mortality was not compared because the unweighted event count in one exposure group was 10 or fewer (Table 3).

**Table 3:** Age-matched comparison of hospital resource utilization in pediatric opioid and cannabis poisoning hospitalizations.

Parameter	Opioid poisoning	Cannabis poisoning	Significance
Total Cases	5,765	5,765	–
Average Age in Years	8.1 (Std. deviation 7.2)	8.1 (Std. deviation 7.1)	*P* = 0.83
Length of stay mean in days	2.5 (Std. deviation 4.8)	1.7 (Std. deviation 1.9)	*P* < 0.01
Total charges mean in $	29,032 (Std. deviation 80,477)	21,364 (Std. deviation 24,336)	*P* < 0.01

Matching was performed on the unweighted discharge records before application of the NIS discharge weights. SMD, standardized mean difference.

### Relative risk of in-hospital complications, opioid vs. Cannabis Poisoning (After Age Matching), Forest Plot

**Figure 3:**
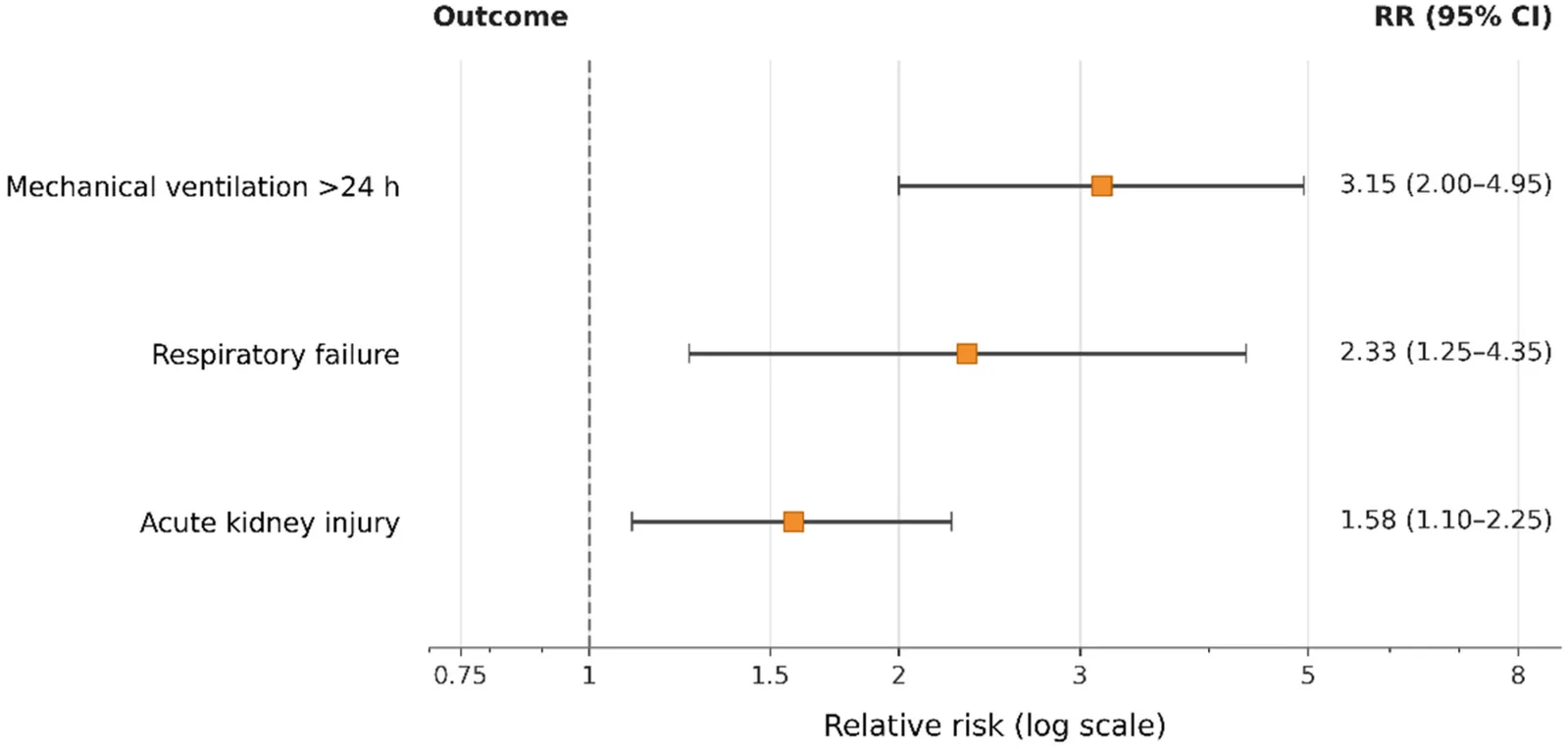
Relative risks of reportable complications associated with opioid vs. cannabis poisoning in the age-matched cohort. Cannabis poisoning served as the reference group. Outcomes with 10 or fewer unweighted events in either exposure group were suppressed.

Figure 3 presents the relative risks of complications that had more than 10 unweighted events in both exposure groups. In the age-matched cohort, opioid poisoning was associated with a higher risk of mechanical ventilation for more than 24 h (RR, 3.15; 95% CI, 2.00–4.95; *P* < 0.001), acute kidney injury (RR, 1.58; 95% CI, 1.10–2.25; *P* = 0.012), and respiratory failure (RR, 2.33; 95% CI, 1.25–4.35; *P* = 0.008). Mortality, acute respiratory distress syndrome, sepsis, pneumonia, urinary tract infection, and mechanical ventilation for more than 96 h were not displayed because at least one exposure-specific unweighted event count was 10 or fewer.

### Multivariable-Adjusted outcomes

In the full unmatched cohort, opioid poisoning remained independently associated with mechanical ventilation for more than 24 h (aOR, 4.65; 95% CI, 2.96–7.31; *P* < 0.001) and acute kidney injury (aOR, 2.55; 95% CI, 1.77–3.69; *P* < 0.001). The adjusted association with respiratory failure did not reach statistical significance after accounting for the NIS survey design and the prespecified covariates (aOR, 1.71; 95% CI, 0.89–3.27; *P* = 0.107).

Opioid poisoning was also associated with an adjusted increase of 1.06 hospital days (95% CI, 0.78–1.34; *P* < 0.001) and $17,286 in total hospital charges (95% CI, $12,752-$21,820; *P* < 0.001). Mortality and other complications with 10 or fewer unweighted events in either exposure group were not included in the regression analysis (Table 4).

**Table 4:** Unadjusted and multivariable-adjusted associations of opioid vs. cannabis poisoning with reportable inpatient outcomes.

Outcome	Unadjusted effect estimate (95% CI)	Adjusted effect estimate (95% CI)	Adjusted *P* value
Respiratory failure	OR 2.28 (1.26–4.12)	aOR 1.71 (0.89–3.27)	0.107
Mechanical ventilation >24 h	OR 5.15 (3.42–7.77)	aOR 4.65 (2.96–7.31)	<0.001
Acute kidney injury	OR 3.59 (2.65–4.86)	aOR 2.55 (1.77–3.69)	<0.001
Length of stay	*β* + 1.29 days (1.04–1.53)	Adjusted *β* + 1.06 days (0.78–1.34)	<0.001
Total hospital charges	*β* + $18,080 ($14,072–$22,088)	Adjusted *β* + $17,286 ($12,752–$21,820)	<0.001

Cannabis poisoning served as the reference group. Adjusted models included age, sex, race, median household income quartile, weekend admission, and admission year and were performed in the full unmatched cohort. Estimates accounted for the NIS discharge weight, sampling strata, and hospital clusters. Outcomes with 10 or fewer unweighted events in either exposure group were not modeled. OR, odds ratio; aOR, adjusted odds ratio; CI, confidence interval; *β*, regression coefficient.

## Discussion

In this national analysis of pediatric inpatient hospitalizations, cannabis poisoning accounted for an increasing proportion of opioid and cannabis poisoning admissions between 2016 and 2022. Nevertheless, opioid poisoning was associated with greater hospital resource utilization and higher risks of selected major complications. These differences remained evident after age matching and multivariable adjustment. Specifically, opioid poisoning was independently associated with mechanical ventilation for more than 24 h, acute kidney injury, a longer hospital stay, and higher hospital charges. The adjusted association with respiratory failure did not reach statistical significance after accounting for the NIS complex survey design.

The opioid-related findings are consistent with previous studies demonstrating substantial respiratory morbidity and critical care utilization among children and adolescents with opioid poisoning (1–4). Opioid toxicity is known to impair central respiratory drive, providing clinical context for the observed association with prolonged mechanical ventilation (13). Opioid-related effects on immune function have also been described; however, the present administrative analysis cannot determine whether such mechanisms contributed to the observed inpatient complications (14). In the age-matched analysis, opioid poisoning was associated with higher risks of mechanical ventilation, acute kidney injury, and respiratory failure. These findings should be interpreted as associations rather than evidence of direct causation because the NIS does not provide detailed clinical information regarding dose, toxicology results, co-ingestions, naloxone administration, or the temporal relationship between poisoning and inpatient complications.

Cannabis poisoning hospitalizations increased substantially during the study period, consistent with previous reports describing increased pediatric exposure to edible cannabis products (5–11). The age distribution demonstrated a considerable burden among children aged 0–2 years and a relative predominance of cannabis poisoning during mid-childhood. These patterns are compatible with prior reports of unintentional ingestion of edible products by young children, although the administrative data used in this study cannot establish the specific product, tetrahydrocannabinol dose, route of exposure, or circumstances of ingestion (12).

The marked adolescent predominance of opioid poisoning may reflect several pathways, including intentional substance use, misuse, self-harm, or polysubstance exposure. However, the available administrative intent codes do not provide sufficient clinical or contextual detail to distinguish these pathways reliably. Therefore, the age-specific findings should be viewed as descriptive patterns that may help identify populations for targeted prevention rather than as evidence of a specific behavioral mechanism.

Opioid poisoning was associated with approximately one additional hospital day and more than $17,000 in additional adjusted hospital charges. These findings indicate a greater hospital charge and inpatient resource burden associated with opioid poisoning and are consistent with previous evidence regarding the economic burden of pediatric opioid poisoning (15). Charges should not be interpreted as actual costs or reimbursements, but their absolute difference remains relevant when considering the comparative hospital resources associated with these admissions.

The findings support complementary rather than competing prevention strategies. For opioid exposures, clinicians and public health agencies may prioritize careful pediatric opioid prescribing, safe storage and disposal, naloxone education, and age-appropriate mental health and substance-use screening (16–19). For cannabis exposures, policymakers, manufacturers, clinicians, and caregivers may support child-resistant packaging, clear labeling, restrictions on youth-appealing product presentation, and secure household storage, particularly for edible cannabis products (5–12).

### Strengths

This study has several strengths. It used a large, nationally representative inpatient database covering seven consecutive years and therefore allowed evaluation of annual changes that could not be examined using the triennial Kids' Inpatient Database. Exposure classification was based on the principal diagnosis, increasing the specificity of the included hospitalizations. The analysis combined age matching with multivariable adjustment and accounted for the NIS discharge weights, sampling strata, and hospital clustering. In addition, both clinical complications and hospital resource-utilization outcomes were evaluated.

### Limitations

This study has several limitations. First, the analysis relied on administrative ICD-10-CM and ICD-10-PCS codes and was therefore subject to coding errors and misclassification of exposure type, complications, co-ingestions, and clinical severity. Exposure groups were defined according to the principal diagnosis, but secondary diagnoses could include additional substances or conditions that contributed to the hospitalization. Second, the NIS is discharge-based rather than patient-based and cannot identify repeat hospitalizations involving the same patient. Third, the database does not include detailed clinical information such as substance dose, route of exposure, toxicology confirmation, naloxone administration, intensive care unit admission, laboratory values, or the timing of complications. Fourth, administrative intent codes do not fully characterize the circumstances of ingestion, including accidental exposure, misuse, intentional substance use, or self-harm. Fifth, although age matching and multivariable adjustment were performed, residual confounding remains possible. Sixth, the NIS was selected to permit annual analyses, whereas the pediatric-specific Kids' Inpatient Database is released only every three years; estimates of rare pediatric outcomes may therefore be less precise in the NIS. Seventh, hospital charges were strongly right-skewed and may have been influenced by a relatively small number of high-charge hospitalizations. Charges were retained on their original scale because the study aimed to estimate the absolute hospital charge burden, but they should not be interpreted as actual costs or reimbursements. Eighth, HCUP reporting requirements prevented comparative reporting and modeling of mortality and other rare outcomes when an exposure-specific unweighted event count was 10 or fewer. Finally, the NIS includes inpatient hospitalizations only and does not represent poisonings treated exclusively in emergency departments, poison centers, outpatient settings, or outside the health care system.

### Practical, policy, and research implications

For clinical practice, the association between opioid poisoning and mechanical ventilation supports careful respiratory assessment and monitoring of hospitalized children and adolescents with suspected opioid exposure. Clinicians caring for children and prescribing opioids can also reinforce safe storage, appropriate disposal, and naloxone education. Public health agencies and health systems may use age-specific prevention strategies, including household exposure prevention for young children and mental health, substance-use, and harm-reduction interventions for adolescents.

For cannabis prevention, policymakers and product manufacturers should consider child-resistant packaging, clear dose labeling, and measures that reduce the resemblance of edible cannabis products to conventional sweets or snacks. Clinicians and caregivers can reinforce secure household storage of all cannabis products.

Future research should use data sources containing toxicology results, product type, dose, co-ingestions, intent, and detailed clinical information. Linkage with poison-center, emergency department, or clinical registry data may be particularly useful for examining rare outcomes, including mortality, that could not be reported reliably in the present analysis.

## Conclusions

Among pediatric hospitalizations with a principal diagnosis of opioid or cannabis poisoning, cannabis poisoning accounted for an increasing proportion of admissions between 2016 and 2022. Opioid poisoning was associated with higher risks of mechanical ventilation for more than 24 h and acute kidney injury, as well as longer hospitalization and higher hospital charges. These findings support respiratory monitoring and opioid harm-reduction efforts by clinicians and public health agencies, together with child-resistant cannabis packaging, safe-storage counseling, and caregiver education. Additional studies with comprehensive clinical characterization are needed to evaluate rare outcomes and determine the relative contributions of poisoning intent, product type, dose, and co-ingested substances to clinical outcomes.

## Data Availability

Publicly available datasets were analyzed in this study. This data can be found here: The data used in this study were obtained from the Healthcare Cost and Utilization Project (HCUP) Nationwide Inpatient Sample (NIS), Agency for Healthcare Research and Quality (AHRQ): https://hcup-us.ahrq.gov/nisoverview.jsp. The NIS data are available through the HCUP Central Distributor after completion of the required HCUP Data Use Agreement training and purchase/application process: https://www.distributor.hcup-us.ahrq.gov/.
